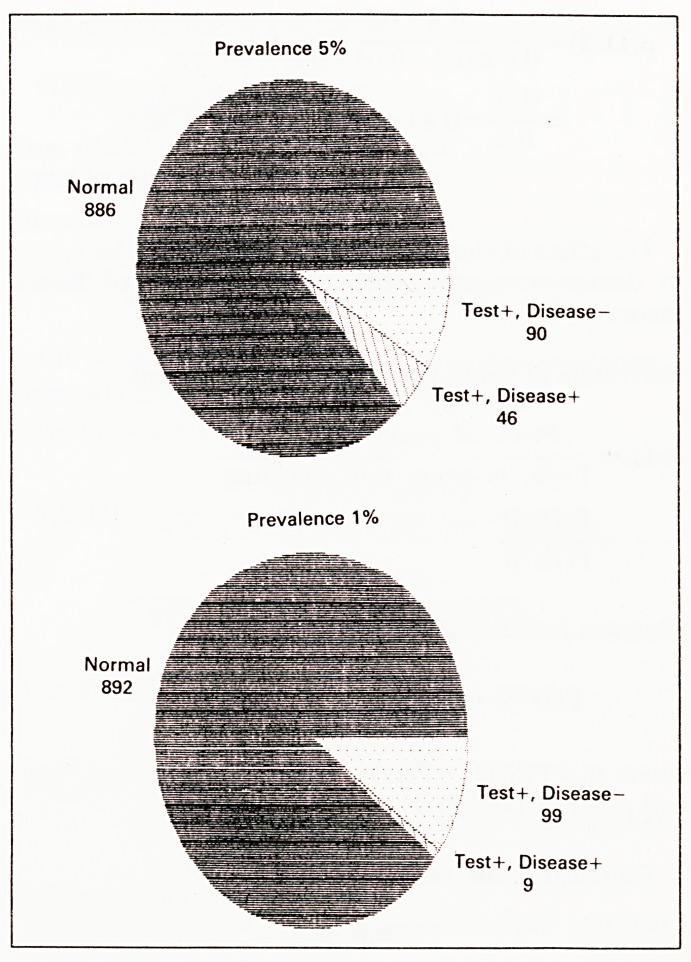# Bayes Theorem for the Clinician

**Published:** 1990-12

**Authors:** G. H. Hall, A. P. Round

**Affiliations:** Consultant Physician, Devon and Exeter Clinical Area; Medical Registrar, Royal Devon and Exeter Hospitals


### Bayes Theorem for the Clinician

**G. H. Hall BSc, MD, FRCP**
Consultant Physician, Devon and Exeter Clinical Area

**A. P. Round MB, ChB, MRCP**
Medical Registrar, Royal Devon and Exeter Hospitals

Osler's injunction to 'diagnose, diagnose, diagnose' can only be justified if we make the correct diagnosis. Quite often we are not sure. We need to be sure enough that the evidence indicates that this is the most likely of the diagnoses considered. We are often denied complete certainty by the constraints of time, expense, difficulty or even danger in collecting all the information we would like. We take into account whether the suspected disease is common or not, and whether the findings tend to support or discount this hypothesis. Such intuitive procedures may be, and often are, seriously biased[1]. It would be preferable to employ an open system where all the assumptions about diagnostic reasoning, and the way in which they are combined, are made quite explicit. Bayes theorem[2] provides such a model. An understanding of how Bayes theorem is derived, its strengths and its weaknesses, greatly enhances both the validity of our own daily practice[3] and our appreciation of the working of diagnostic machines.

**Bayesian thinking in Diagnosis**

A diagnosis is favoured if the disease (D) is common and the symptoms (S) are characteristic. The basic question is: given S, how likely is D? Under consideration there are usually many symptoms—$S_1, S_2, \ldots S_i$, and many diseases—$D_1, D_2, \ldots D_j$. So a particular example might be: how likely is $D_3$ given $S_2$? S may consist of a group of symptoms, say $S_1$ and $S_3$ and $S_7$ The term symptom will be taken to mean any finding—(symptom, sign, test result, etc.

**How common is the disease?**

In the calculations which follow the prevalence or frequency of a condition is taken as equivalent to its probability. If a population contains N individuals, and there are n(D) people with the disease D, then

$$P(D)=\frac{n(D)}{N}$$

where P(D) is the probability of the disease D. Probability is expressed as a fraction of 1, rather than as a percentage. The idea of probability includes expectation as well as frequency. Thus we expect a large number of tosses of a coin to produce about half heads and half tails.

Likelihood and expectation can also be represented by the odds ratio. Although a familiar term from betting in horses, it is not an easy concept. The odds of an event occurring is the ratio of the probability of the event occurring to the probability of the event not occurring. For example, the probability of throwing a six at dice is 1/6, and of not throwing a six is 5/6. Then the odds of throwing a six are $\frac{1/6}{5/6}=1{:}5$. The odds of having the disease D are $P(D)/P(\bar{D})$ where $\bar{D}$ means the absence of D. Since it is certain (P = 1) that a person either has the disease or does not, then

$$P(D)=1-P(D) \text{ and odds } D=\frac{P(D).}{1-P(D)}$$

**How characteristic are the symptoms?**

For a symptom to be useful in diagnosis it must be more common in those with the disease in those than without it. The symptom frequency, denoted P(S/D) is the ratio of the number of people with the symptom and the disease, n(S&D), to the number of people with the disease, n(D).

$$p(S/D=\frac{n(S\&D)}{n(D)}$$ This is the same as the <u>sensitivity</u> of a test or <u>true positive rate.</u>

Likewise, the symptom frequency in those without the disease is

$$p(S/\bar{D})=\frac{n(S\&\bar{D})}{n(\bar{D})}$$ This is the same as the <u>false positive rate.</u>

The <u>specificity</u> of a symptom in a disease is the ratio of the number of people without the symptom and without the disease to the number of people who do not have the disease.

$$\frac{n(\bar{S}\&\bar{D})}{n(\bar{D})}$$

and as all people without the disease either have the symptom or do not

$$n(S\&\bar{D})+n(\bar{S}\&\bar{D})=n(\bar{D})$$

Dividing both sides by $n(\bar{D})$

$$\frac{n(S\&\bar{D})}{n(\bar{D})}+\frac{n(\bar{S}\&\bar{D})}{n(\bar{D})}=1$$

Therefore

$$\frac{n(S\&\bar{D})}{n(\bar{D})}=1-\frac{n(\bar{S}\&\bar{D})}{n(\bar{D})}$$

False positive rate = 1 − specificity.

When a symptom is common in a disease and rare in the rest of the population, its diagnostic value is high.

This diagnostic value can be expressed as a likelihood ratio (L.R.).

$$\text{L.R.} = \frac{\text{True positive rate}}{\text{False positive rate}} = P(S/D):P(S/\bar{D})$$

or

$$\frac{\text{sensitivity}}{1-\text{specificity}}$$

Example: It was found in a particular study that 80% of patients with a pulmonary infarct had pleuritic pain $p(S/D)=0.8$, whereas only 5% of the other patients had it $p(S/\bar{D})=0.05$. Then $\text{L.R.} = \frac{0.8}{0.05} = 16:1$

Put another way, patients with a pulmonary infarct were 16 times more likely than other patients to have pleuritic pain.

**Predictive value of a Symptom**

It is often thought that the sensitivity and the specificity of a test, as combined in the LR, are all that is necessary to interpret a positive finding and apply it to the patient. This is incorrect: the prevalence of the suspected disease must be taken into account.

We want to know the probability of the disease given the symptom—let us call this P(D/S).

This will be

$$\frac{n(S\&D)}{n(S\&D)+n(S\&\bar{D})}$$

or

$$\frac{\text{number of true positives}}{\text{number of true positives} + \text{number of false positives}}$$

This is the predictive value of a test.

Example: Suppose the frequency of rheumatoid arthritis in a population is 5% $P(D)=0.05$, $P(\bar{D})=0.95$.

The sensitivity of the RA flocculation test is 90%. $P(S/D)=0.9$ and the specificity is 90% ($P(S/\bar{D})=1-\text{specificity}=1-0.9=0.1$). Then in a sample of 100 people.

$n(S\&D)=5\times0.9=4.5$ or TRUE POSITIVES

$n(S\&\bar{D})=95\times0.1=9.5$ or FALSE POSITIVES.

There are 14 (9.5+4.5) people with a positive RA test, and of these only 4.5 have the disease. The predictive value of a positive test is therefore only

$$\frac{4.5}{14}=0.32 \text{ (see pie charts)}$$

Compare this with the sensitivity

$p(S/D)=0.9$ and L.R. 9:1

Both are much greater than the predictive value of a positive finding because they omit consideration of the low incidence of the disease in the population.

Bayes theorem itself takes the general form of the predictive value ie.

$$\frac{TP}{TP+FP} \text{ (this is in absolute numbers, not rates)}$$

We seldom know the exact number of true positive and false negative findings in a population: these have to be estimated from samples.

By deriving a formula (see appendix A) one can utilise true and false positive rates together with the prevalence of the disease to calculate the probability of disease given the symptom i.e.

$$p(D/S)=\frac{p(S/D)\times p(D)}{p(S/D)\times p(D)+p(S/\bar{D})\times p(\bar{D})}$$

$$=\frac{\text{TP rate}\times\text{prevalence}}{\text{TP rate}\times\text{prev.}+\text{FP rate}\times(1-\text{prev.})}$$

The Bayes calculation shows how the prior probability of a disease p(D) is altered to a posterior probability p(D/S) given a new piece of evidence S.

Example: In the previous example suppose we know that the prevalence of pulmonary infarcts in our population is 20%. A patient comes in with pleuritic pain. What is the chance that this patient now has a pulmonary infarct?

True positive rate = 0.8
False positive rate = 0.05.
Prevalence = 0.2

$$p(D/S) = \frac{0.8 \times 0.2}{0.8 \times 0.2 + 0.05 \times 0.8}$$

$$= \frac{0.16}{0.2} = 0.8$$ i.e. there is now a 80% chance that this patient has a pulmonary infarct.

The effect of changing the probabilities may be easier to demonstrate using the "odds" version of Bayes theorem.

**Derivation of Odds Version of Bayes theorem.**

$$\text{Odds} = \frac{\text{Prob. of event occurring}}{\text{Prob. of event not occurring.}}$$

$$= \frac{P(D/S)}{P(\bar{D}/S)}$$

Classical Bayes is of the form

$$P(D/S) = \frac{p_1}{p_1 + p_2} \text{ and } P(\bar{D}/S) = \frac{p_2}{p_1 + p_2}$$

where $p_1 = P(S/D) \times P(D)$

and $p_2 = P(S/\bar{D}) \times P(\bar{D})$

$$\text{Therefore odds ratio} = \frac{p_1}{p_1 + p_2} \times \frac{p_1 + p_2}{p_2}$$

$$= p_1/p_2$$

$$= \frac{P(S/D) \times P(D)}{P(S/\bar{D}) \times P(\bar{D})}$$

Now

$$\frac{P(S/D)}{P(S/\bar{D})} = LR$$

and

$$\frac{P(D)}{P(\bar{D})} = \text{prior odds of disease.}$$

Therefore Posterior odds = prior odds × LR.
Example: We found that, in the previous example, the LR for pleuritic pain in pulmonary infarction was 16:1. Suppose we know that the prevalence of pulmonary infarction was 20% in the study (prior odds 1:4). Given the finding of pleuritic pain, the posterior odds

$$= \frac{1}{4} \times \frac{16}{1} = \frac{4}{1}$$

Note that these odds are the same as the 80% probability calculated in the classical way.

**Incorporation of additional evidence**
Suppose a further test e.g. V/Q scanning with an LR of 4:1[4] was performed and found to be positive. The posterior odds calculated previously have now become the prior odds and the new result is

$$\text{Posterior odds} = 4:1 \times 4:1$$

$$= 16:1$$

**Logarithmic form of odds ratio**
Every additional test involves a further multiplication by the appropriate LR.

$$\text{Post. odds} = \text{prior odds} \times LR_1 \times LR_2 \times LR_3 \ldots LR_n$$

Taking logarithms,

$$\text{Log. post. odds} = \text{Log. prior odds} + \log. LR_1 + \log. LR_2 \ldots LR_n$$

In the example, using logs. to base 2:

$$\text{Log}_2 \text{ post. odds} = -2 + 4 + 2$$
$$= 4$$
$$\text{Posterior odds} = 2^4 = 16:1$$

The usual form of log. transformation is to use $\log_{10}$ and multiply by 10 to give a whole number score. Probability may be obtained from the odds by the formula P = odds/1 + odds.

**The problem of interdependence**
Sometimes different tests are measuring the same thing, so simply applying LR's in succession may exaggerate the final odds. As an extreme example suppose it was the same individual patients who had positive results in two tests. (For this to be so, the tests would have to have the same P(S/D)). Clearly the second test has provided no new information, yet the odds will have increased. Although test results are seldom correlated completely like this, one expects some degree of concomitant variation because they are chosen to detect the same thing, namely, the hidden disease.

Some shrinkage factor may need to be applied to correct the overestimate of posterior odds (e.g. by logistic regression, ridge regression or principal components analysis), though when the number of tests is small this is not usually necessary.

**Negative results**
These may be dismissed as non-contributory, but such results can be used in the same way as positive results. Thus the LR for a negative test would be

$LR(-) = P(\bar{S}/D)/P(\bar{S}/\bar{D})$
$P(\bar{S}/D)$ is the false negative rate.
$P(\bar{S}/\bar{D})$ is the true negative rate.

Tests seldom confirm or refute a diagnosis completely, but they may support or discount it, i.e. they increase or decrease the prior odds.

**Using Posterior Odds**
The relative probability of only two mutually exclusive and comprehensive outcomes can be calculated by an application of Bayes formula. Where there are several possibilities, each can be compared with the rest, in the light of the evidence obtained, and the disease with the highest posterior probability chosen. The reliance one would place on a result naturally would depend on the confidence limits of the original data, e.g. were the prior odds obtained from one's own population? Were the numbers large enough? Were the observers or test methods different from the original example?

When a defined figure for probability of a diagnosis is available, it is immediately obvious when a previously determined threshold level of probability for action has been exceeded. The threshold level of probability for action (i.e. the levels where the pros and cons of treatment or investigation are equally balanced) can be shown to be

$$\frac{1}{(B/C)+1}$$

when B = benefit and C = cost.

Thus, suppose the relative benefit to cost of anticoagulation is 3:1 in pulmonary infarction, then the treatment threshold of probability would be $\frac{1}{3+1}=0.25$. It would be unnecessary to demand more tests if the initial Bayesian calculation showed the P(D) exceeded 0.25 (other things being equal).

Another advantage of having a number for diagnostic probability, rather than an adjective, is that one can choose the best line of action to pursue. Sometimes it is difficult to decide whether to select a diagnosis of low probability because the condition is easily treatable, rather than a more probable diagnosis where the treatment may be less effective or dangerous. The "best diagnostic value" will come from the sum of the products of the diagnostic probabilities and utilities of each outcome. This is what we do intuitively and often incorrectly[1]. A precise value for one of the variables (probability) does provide a more plausible and assured indication of what best to do.

Often too much credence is paid to test results, especially when an arbitrary line is drawn between normal and abnormal results. "Reference ranges" are usually provided only for "normal" values, and give no idea of the possible overlap with the reference ranges for diseased groups. Altering the cut-off points will alter the TP/FP ratios and hence the likelihood ratios. Mis-classification may lead to missed diagnoses or unnecessary treatment—and the lesser of two evils may be selected by appropriate choice of the cut-off points.

Diagnostic machines often rely explicitly or implicitly on a Bayesian model. An understanding of Bayesian principles allows a better appreciation of the strengths and weaknesses of these aids.

**Derivation of Bayes theorem**

We need to find P(D/S)

$$P(D/S)=\frac{n(S\&D)}{n(S)}\ldots 1$$

$$\text{We know } P(S/D)=\frac{n(S\&D)}{n(D)}\ldots 2$$

$$\text{From 1, } n(S\&D)=P(D/S)\times n(S)\ldots 3$$

$$\text{From 2, } n(S\&D)=P(S/D)\times n(D)\ldots 4$$

$$\therefore\ P(D/S)\times n(S)=P(S/D)\times n(D)$$

$$P(D/S)=\frac{P(S/D)\times n(D)}{n(S)}$$

Now $n(S)=n(S\&D)+n(S\&\bar{D})$

$$P(D/S=\frac{P(S/D)\times n(D)}{n(S\&D)+n(S\&\bar{D})}$$

Now $n(S\&D)=P(S/D)\times n(D)\ldots 4$

Likewise $n(S\&\bar{D})=P(S/\bar{D})\times n(\bar{D})$

Therefore

$$P(S/D)=\frac{P(S/D)\times n(D)}{P(S/D)\times n(D)+P(S/\bar{D})\times n(\bar{D})}$$

Let N be the number of individuals in the population.

Then n(D)/N = the prevalence of the disease.

Divide top and bottom by N.

$$P(S/D)=\frac{P(S/D)\times n(D)/N}{P(S/D)\times n(D)/N+P(S/\bar{D})\times n(\bar{D})/N}$$

$$=\frac{P(S/D)\times P(D)}{P(S/D\times P(D)+P(S/\bar{D})\times P(\bar{D})}$$

or as stated previously

$$\frac{\text{True pos. rate}\times\text{prevalence}}{\text{True pos. rate}\times\text{prevalence}+\text{False pos. rate}\times(1-\text{prevalence})}$$

**REFERENCES**

1. KIRWAN, J. R. *et al*. Clinical Judgement Analysis. *QJM* 1990; **76**: 935–949.
2. BAYES, T. An Essay towards solving a problem in the doctrine of chances. *Biometritika* 1758; **45**: 296–315.
3. HALL, G. H. The clinical application of Bayes theorem. *Lancet* 1967 ii; 555–557.
4. GRAY, H. W. *et al*. Lung scanning for pulmonary embolism: clinical and pulmonary angiographic correlations. *QJM* 1990; **77**: 1135–1150.

## Figures and Tables

**Figure f1:**